# The Mechanism of Fracture and Damage Evolution of Granite in Thermal Environment

**DOI:** 10.3390/ma14237234

**Published:** 2021-11-26

**Authors:** Suran Wang, Youliang Chen, Min Xiong, Xi Du, Guanlin Liu, Tomás Manuel Fernández-Steeger

**Affiliations:** 1 Department of Geotechnical Engineering, College of Civil Engineering, Tongji University, Shanghai 200092, China; evanwangsuran@foxmail.com (S.W.); 1310298@tongji.edu.cn (M.X.); 2 Department of Civil Engineering, University of Shanghai for Science and Technology, Shanghai 200093, China; duxijl@163.com (X.D.); liuguanlin60119@163.com (G.L.); 3 Department of Engineering Geology and Hydrogeology, RWTH Aachen University, 52064 Aachen, Germany; 4 Institut für Angewandte Geowissenschaften, Technische Universität Berlin, 10587 Berlin, Germany; fernandez-steeger@tu-berlin.de

**Keywords:** thermal effect, mechanical properties, shear cracks, tensile cracks, Voronoi method

## Abstract

In the study of rock mechanics, the variation of rock mechanical characteristics in high-temperature environments is always a major issue. The discrete element method and Voronoi modeling method were used to study the mechanical characteristics and crack evolution of granite specimens subjected to the high temperature and uniaxial compression test in order to study the internal crack evolution process of granite under the influence of high temperatures. Meanwhile, dependable findings were acquired when compared to experimental outcomes. A modified failure criterion was devised, and a Fish function was built to examine the evolution behavior of tensile and shear cracks during uniaxial compression, in order to better understand the evolution process of micro-cracks in granite specimens. Shear contacts occurred first, and the number of shear cracks reached its maximum value earliest, according to the findings. The number of tensile contacts then rapidly grew, whereas the number of shear cracks steadily declined. Furthermore, it was found that when temperature rises, the number of early tensile cracks grows. This study develops a fracture prediction system for rock engineering in high-temperature conditions.

## 1. Introduction

Deterioration of the mechanical properties of crystalline rocks due to temperature variations is always a problem in the field of rock mechanics, as thermal attack induces new microcracks or enlarges existing microcracks. The physical and mechanical properties of rocks after heat treatment have been investigated extensively [1,2,3,4,5,6,7], since brittle rocks usually have a complicated mechanical behaviour related to their internal microstructure [8,9]. Microstructural changes, especially the development of microcracks, affect their mechanical properties [10,11].

Along with the experimental investigations of the mechanical properties of rocks, extensive numerical studies have also been conducted to simulate rock behaviour. The discrete element method (DEM) has been used extensively for the numerical simulation of rock and rock mass, as the finite element method is not able to model the development of cracking and slope failure by slip. Abe (2016) analysed the elastic properties, such as the Young’s modulus and Poisson’s ratio of a DEM material, and found that the influence of damage on the Poisson’s ratio and Young’s modulus depends on the coefficient of friction between crack surfaces under compression [12]. Li and Konietzky (2014) investigated time-dependent crack growth, and in their model, the damage process and macroscopic fracture pattern were simulated based on the theory of linear elastic fracture mechanics [13].

The grain-based method (GBM) has also recently been used to study the mechanical properties of rocks. Bahrani and Kaiser (2016) used GBM to investigate the influence of specimen size on the strength of intact rocks and rocks with defects in unconfined conditions [14]. They found that the strength of the specimens with defects either decreased, increased, or fluctuated with increasing specimen size depending on the orientation of the defects relative to the loading direction. Meanwhile, the particle flow code is also an effective method to investigate the mechanical characteristics of rocks. Zhang et al. (2014) simulated a biaxial compression test using the bonded particle model (BPM) within the particle flow code (PFC2D) [15]. Their results showed that there are three main stages of micro-crack development: the initial stable development stage, the rapid increase stage, and the final stable development stage. Zhang et al. (2016) carried out numerical simulations of Brazilian tests and uniaxial compression tests at different loading rates [16]. The results indicated that in both tests, acoustic emission and strain energy in the specimens increased nonlinearly with an increasing loading rate. As mentioned above, many studies have been carried out using the discrete element method to examine several factors. However, most authors have used PFC for their numerical simulations. A current limitation of PFC is that it significantly overestimates the tensile strength of rock-type materials when the models have been calibrated based on unconfined compressive strength [17,18,19].

A solution to the problem of obtaining a realistic ratio of unconfined compressive strength (UCS) to tensile strength was proposed for use in Universal Distinct Element Code (UDEC) [20]. A list of discrete element and hybrid finite discrete element modeling methodologies for simulating fracture processes in rocks and rock masses was offered in the field of rock mechanics. [21]. Gao et al. (2016) simulated the microstructure of rock-like materials using GBM in UDEC [22]. Their results show that the influence of shear cracking dominates over tensile cracking under axial compression at the UCS, which agrees with the results reported [1]. In contrast, Nicksiar and Martin (2014) believe that only tensile cracks appear at crack initiation. Therefore, the mechanisms of fracture initiation are worth exploring [23].

Thermal damage should also be considered in numerical simulations. A coupled thermo-mechanical model can be built using the combined finite-discrete element method (FDEM). A thermal cracking example has previously been assessed and model predictions compared with experimental results verifying the correctness of the coupled model in dealing with the problem of thermal cracking [24]. However, the mechanical response of rock-type materials, such as their stress-strain curve and uniaxial compressive strength, should be investigated further by means of thermo-mechanical calculation.

In this work, a novel approach was used to investigate the discreteness of rock materials after thermal treatment using the Voronoi method in UDEC. The findings related to mechanisms of fracture initiation and the relationship between shear cracking and tensile cracking will be discussed. The mechanical characteristics of rock specimens tested after high-temperature exposure in a laboratory were used to investigate the macroscopic and microscopic mechanical behavior of rock specimens from the Fujian province, China. The result provides a significant method and effective parameters for rock engineering subjected to high temperatures.

## 2. Theoretical Basis

In this study, the Voronoi method was selected to build a random joint model with a width of 50 mm and a height of 100 mm. How cracks appear and develop was studied by observing changes in the modelled contacts.

In addition, “fully deformable” blocks were used in the UDEC model which allowed for internal deformation of each block. Although early discrete element algorithms believed that blocks were rigid, the importance of block deformability is increasingly acknowledged, notably in stability assessments of subterranean entrances and seismic response models of buried structures. In this study, deformable blocks were chosen to model the granite specimens after high-temperature exposure.

## 3. Numerical Model

### 3.1. Model Setup

**Figure 1:**
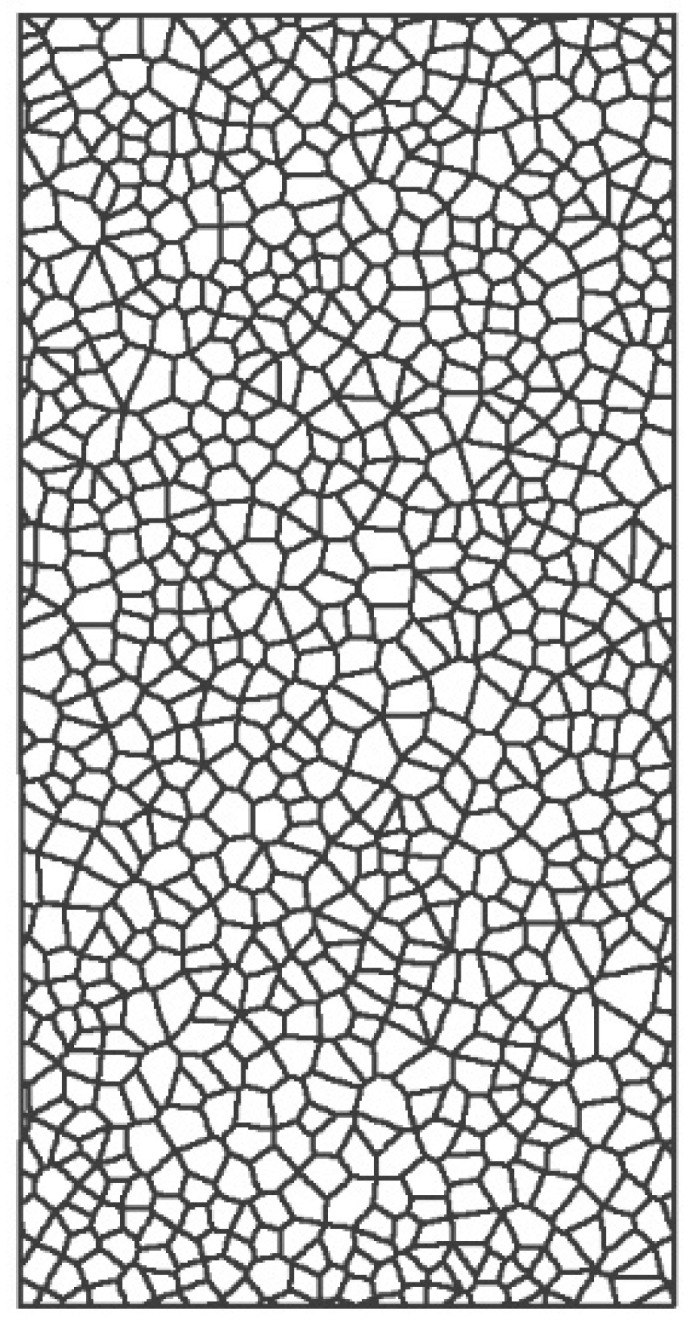
Numerical model with two block edge sizes of 2.5 mm.

Models with a block edge size of 2.5 mm were built as shown in Figure 1. Fractures appear only at the joints in the model as they are generated when the contact surfaces separate. Due to the random joint arrangement and the lack of porosity in the numerical model, the number of contacts is controlled by the block edges. Based on these modelling conditions, the following results can be expected: (1) The location of crack initiation will be random; (2) a sample modelled in UDEC will have several failure modes; (3) the UCS of the sample in UDEC will be affected by the discreteness; and (4) the block edges in the model will affect the results of the simulation.

### 3.2. Block Constitutive Model

Crack initiation in the laboratory specimens occurred well before the UCS was reached. In the numerical simulation in UDEC, cracking at low loads was expected to occur along the contact surfaces as the blocks deform elastically. Consequently, an elastic model with thermal parameters was selected to model the elastic response of the granite material. The parameters required for the model include the density, bulk modulus, shear modulus, thermal conductivity, specific heat, and thermal expansion. The density, bulk modulus, and shear modulus were obtained from experimental results which have already been published [4]. The thermal parameters used were taken from the Chinese Thermal Design Code for Civil Buildings GB50176-93, 1993, see in Table 1. The granite used in the study was collected from an outcrop located in Nan′an City, Fujian Province, China, from a depth of 2 m. The diameter and the height of the specimens were 50 mm and 100 mm, respectively, in line with the recommendations of the International Society for Rock Mechanics (ISRM).

### 3.3. Contact Constitutive Model

**Figure 2:**
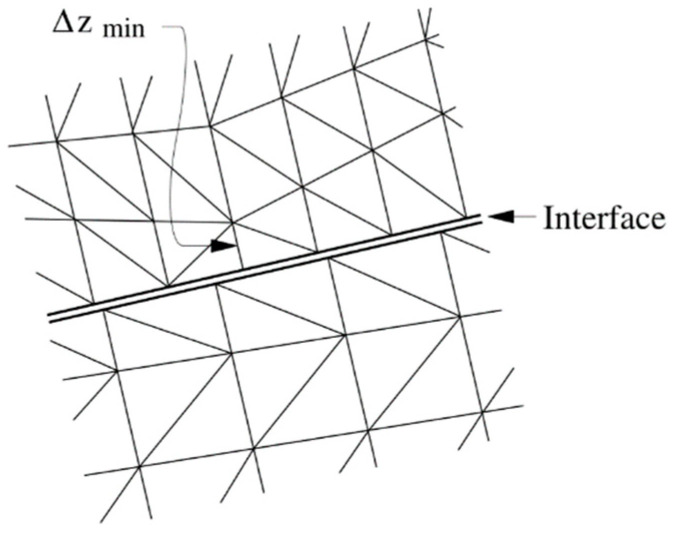
Dimension of zone used in stiffness calculation.

The contact behavior was described using a Coulomb slip model with residual strength properties. The values for cohesion and friction at initial contact were based on the results of a tri-axial compression test and were modified for use in UDEC. It was assumed that contact locations lose their cohesion and friction when the failure occurs. Values for the normal and shear stiffnesses of the contacts were calculated using the method proposed in the UDEC manual. The equation for calculating the fictitious joint normal stiffness and the joint shear stiffness from the equivalent stiffness, expressed in stress-per-distance units of a zone, is of the following form [25]:

(1) $$k_{n}=k_{s}=f\times \max\left[\frac{K+\frac{4}{3}G}{{\Delta Z}_{\min}}\right]$$

where *f* is a multiplication factor, usually set as 10; K and G are the bulk and shear moduli, respectively; and ∆Z_min_ is the smallest width of an adjoining zone in the normal direction, see Figure 2; the “max []” notation indicates that the maximum value overall zones adjacent to the joint is to be used, there may be several materials adjacent to the joint. The parameters for the modelled joints were calculated using Equation (1) and modified prior to use in the model, see Table 2.

### 3.4. Calibration

**Figure 3:**
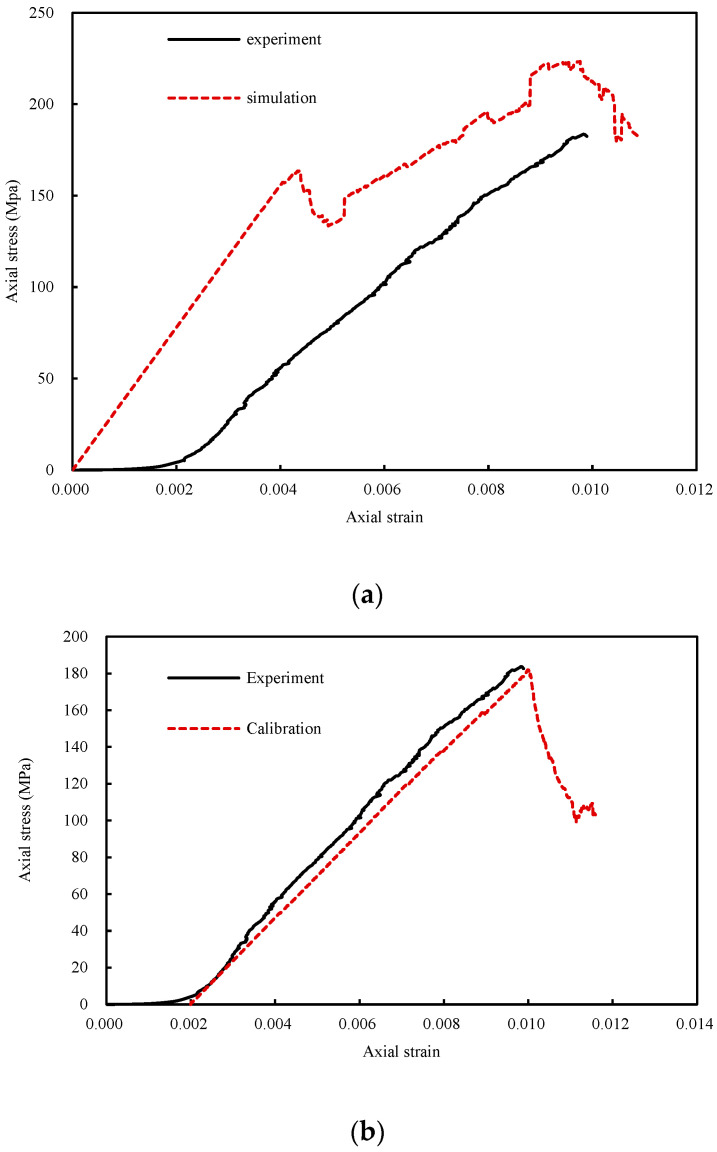
The strain-stress curves: (**a**) before calibration; (**b**) after calibration.

The simulated results of the uniaxial test in UDEC before and after calibration are shown in Figure 3, and the parameters before and after calibration are shown in Table 1 and Table 2. The data of experiment curves and the experimental test method were obtained from Chen et al. (2017) [4]. Several phenomena are illustrated in the Figures: (1) The simulated curves have no compaction stage and started from the elastic stage. Therefore, the simulated curves are translated to the endpoint of the elastic stage of experimental curves for calibrating the parameters; (2) the simulated curves have obvious stable crack developing stage and unstable crack developing stage; (3) the elastic modulus before calibration is higher than that after calibration. However, due to the calculation of the crack developing stage, the results before and after calibration are similar. If the strain is recorded from 0 after calibration, the peak strain will be the result of a lack of the compaction stage strain; and (4) when the experimental Young′s modulus is selected to calculate the joint parameters, the simulated peak stress is larger than the experimental result. When the experimental elastic modulus is selected to calculate the joint parameters, the simulated peak strain is smaller than the experimental result.

## 4. Results and Discussion

### 4.1. Stress-Strain Relationship of Granite Specimens after Thermal Damage

**Figure 4:**
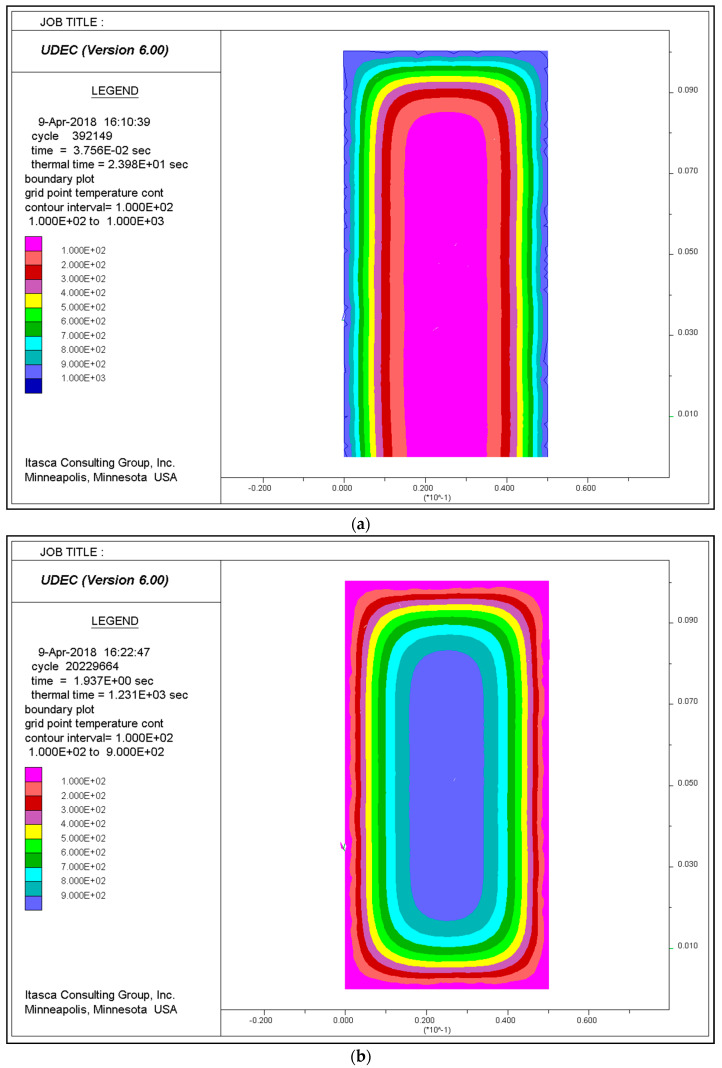
The process of: (**a**) heating; (**b**) cooling down.

The stress-strain relationship for the uniaxial compression of granite after thermal damage was examined in two ways. The results for the 1000 °C thermal damage sample will be used as an example to explain the two methods here. In Figure 3, the “1000 °C experimental result” curve corresponds to the stress-strain relationship of granite after heating to 1000 °C and cooling to room temperature in a furnace, followed by uniaxial compression testing in a laboratory. The other curve corresponds to UDEC predictions. To model the loading during the uniaxial compression test, the UDEC calculations employed a constant boundary velocity. The curve labelled “1000 °C thermo-mechanical result” is from an analysis which uses the experimental values for the mechanical parameters of untreated granite. These mechanical parameters were also taken from laboratory uniaxial compression and triaxial compression tests. In this model, the analysis includes an initial thermal loading cycle from 20 °C to 1000 °C and back down to 20 °C before the uniaxial compression load is applied (Figure 4). Values for the mechanical parameters used in the analyses are shown in Table 3.

**Figure 5:**
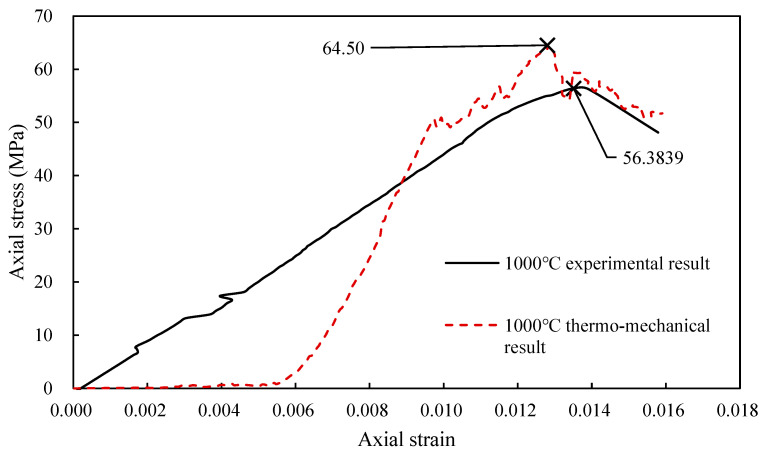
Comparison of experimental and predicted stress–strain relationships for rocks subjected to heat treatment at 1000 °C.

UCS measured 56.39 MPa in the lab, while expected values were 64.50 MPa (shown in Figure 5). The experimental value was lower, and the value from the “1000 °C thermo-mechanical result” model was higher. Despite the tiny amount of inaccuracy, the results should be highlighted. Because the choice of input parameters can affect the results, the measured value of UCS is lower than the predicted value. Because there are so many variables to consider, there is a higher probability of making a mistake. In general, the predicted result in UDEC matched the experimental result.

### 4.2. Stress-Strain Curves for Granite after Heat Treatment at Different Temperatures

**Figure 6:**
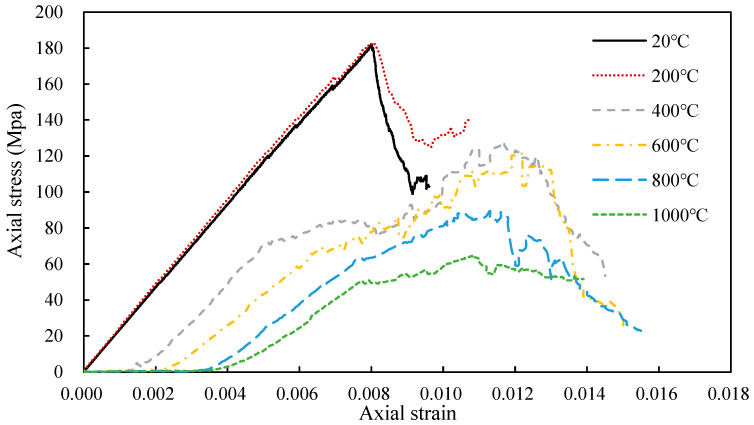
Predicted stress-strain relationship for uniaxial compression after calculation of thermal cycle-induced stress.

Figure 6 shows the simulated stress-strain relationship for granite under uniaxial compression after thermal exposure at different temperatures. The overall trends in the stress–strain data from UDEC are similar to the laboratory results as in both cases, and the stress which can be sustained by the granite decreases with an increase in heat treatment temperature. After exposure to temperatures of 400 °C and above, the compaction stage becomes more distinct and longer with increasing temperature and the gradient of the curve decreases. The curves are also smoother after heat treatment at higher temperatures.

In the analysis, the difference arises because the joints in the models are different. Discreteness is modelled using the Voronoi method in the models, and the different distribution of elements within each model influences the calculation results. The results for the 1000 °C models are very similar to the laboratory results (Figure 5). The thermo-mechanical calculation in UDEC provides a valuable assessment method for rock engineering, which was exposed to high temperatures.

### 4.3. Comparison between High-Temperature State and Cooled State

**Figure 7:**
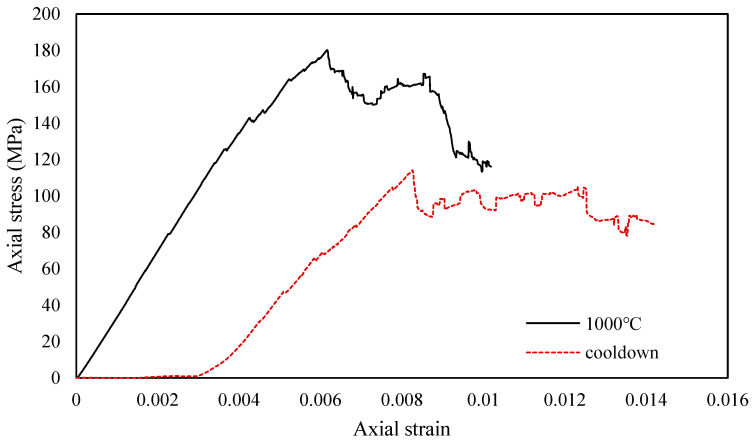
Stress-strain relationship for rock at 1000 °C and post cool-down (reprinted from [26] with permission of Springer Nature).

**Figure 8:**
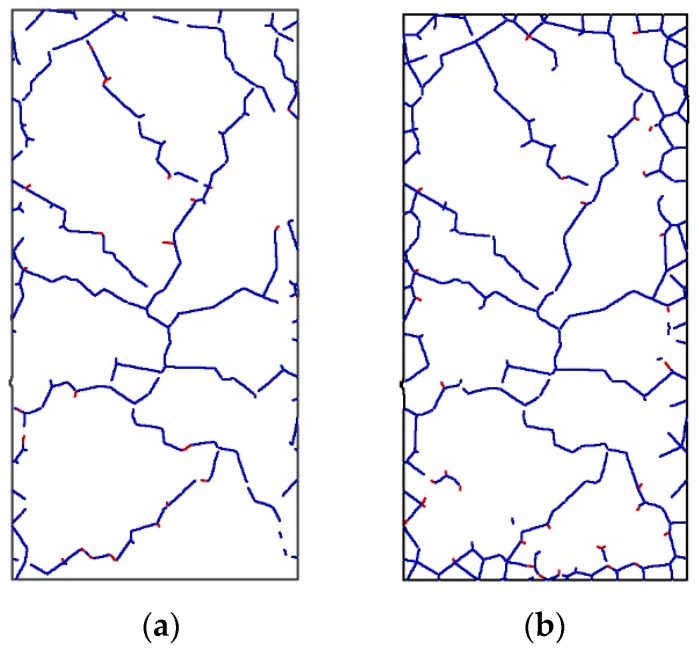
Microcracks in rock specimens: (**a**) at 1000 °C; (**b**) after cool-down to 20 °C.

Thermal damage causes thermal stress in a rock-type material. In a rock-type material, thermal damage creates thermal stress. The high temperature and post-cooling behavior of rocks will be compared using the 1000 °C heating cycle as an example. The estimated stress–strain relationships for granite at 1000 °C and granite cooled to room temperature are shown in Figure 7. There is a significant difference between these two curves. After cooling to room temperature, the elastic modulus falls and the strain at peak stress rises. After cooling down, the strain and UCS are roughly 134.1% and 63.7% of what they were at 1000 °C, respectively. The mechanical characteristics of a specimen are affected by thermal stress after heat treatment at 1000 °C. Pictures of the microcracks within the specimen at 1000 °C and after cool-down are shown in Figure 8. It can be seen that during cool-down, the volume of the specimen decreases and microcracks develop near the boundary. As these microcracks develop, the rock becomes much easier to break, and this microcracking at the boundary is one of the main reasons for the observed decrease in UCS after high-temperature exposure.

### 4.4. Shear Cracking and Tensile Cracking

**Figure 9:**
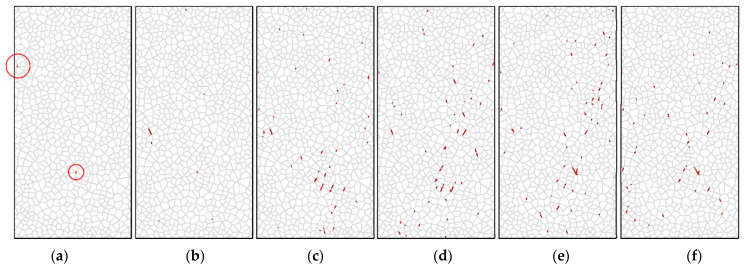
Development of shear cracks in rock specimen in UDEC: (**a**) step 30,000; (**b**) step 60,000; (**c**) step 90,000; (**d**) step 120,000; (**e**) step 150,000; (**f**) step 180,000.

**Figure 10:**
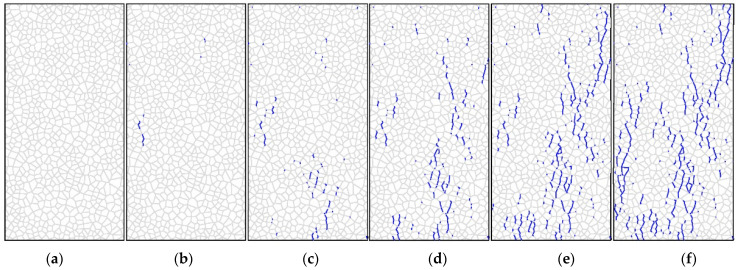
Development of tensile cracks in rock specimen in UDEC: (**a**) step 30,000; (**b**) step 60,000; (**c**) step 90,000; (**d**) step 120,000; (**e**) step 150,000; (**f**)step 180,000.

Figure 9 and Figure 10 show the model predictions for the development of shear cracks and tensile cracks during unconfined uniaxial compression loading. Figure 9a–c and Figure 10a–c correspond to the stage before the UCS has been reached, while Figure 9d and Figure 10d correspond to the sample reaching the UCS. Figure 9e,f and Figure 10e,f show the crack distribution after failure. Only tensile cracks occur during crack initiation, according to Nicksiar and Martin (2014), who employed UDEC-GBM with unbreakable grains to model hard crystalline rocks [23]. However, Figure 9a shows that the initial cracks predicted by this model are shear cracks, indicated by the red circle.

**Figure 11:**
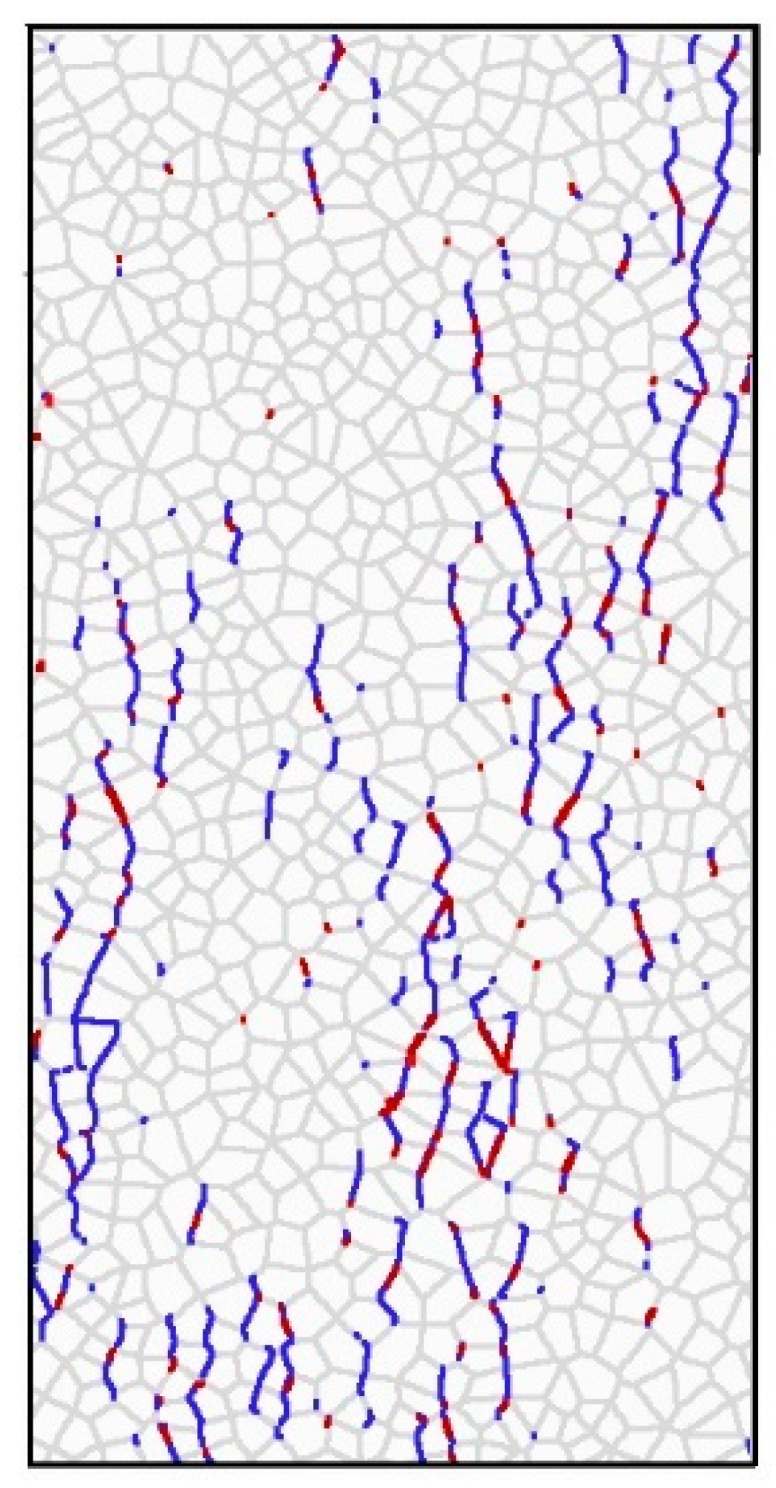
Comparison between shear cracking and fractures.

Figure 11 is a combination of Figure 9a–f and Figure 10f. The shear cracks are shown in red and the tensile cracks are shown in green. Due to the judging criteria for contact failure contained in UDEC, the shear cracks can only be seen at certain times. As shown in Figure 11, most of the shear cracks change to tensile cracks in UDEC where the shear cracks and tensile cracks meet.

According to Gao et al. (2016), the failure status of a contact at a specific loading stage in the UDEC Graphical Interface is dependent on the stress across the contact at that moment [27]. As a result, a contact that fails in shear at first may later be classified as a tensile crack. The contact evaluation criterion in UDEC can be described as follows.

If the shear force of a joint equals the shear limit, then the contact is regarded as a shear crack. If the normal force or shear force of a joint equals zero, then the contact is regarded as a tensile crack. Once contact is considered to have undergone shear failure or tensile failure, fracture occurs.

It is unsuitable to use this criterion to model shear cracking, as the contact is no longer considered to be a shear contact once the shear force exceeds the limit. Therefore, a modified criterion is proposed in this paper in which contacts which meet the condition of Equation (2) are deemed to be shear cracks,

(2) $$\left|\tau_{s}\right|\geq c+\sigma_{n}\tan\varphi =\tau_{\max}$$

where *τ*_s_ is the shear stress, c is the cohesion, and φ is the frictional angle. Under this new criterion, contacts with a shear force greater than or equal to the shear limit *τ*_max_ are recorded as shear cracks. If the normal force of a joint equals zero, then the contact is regarded as a tensile crack.

**Figure 12:**
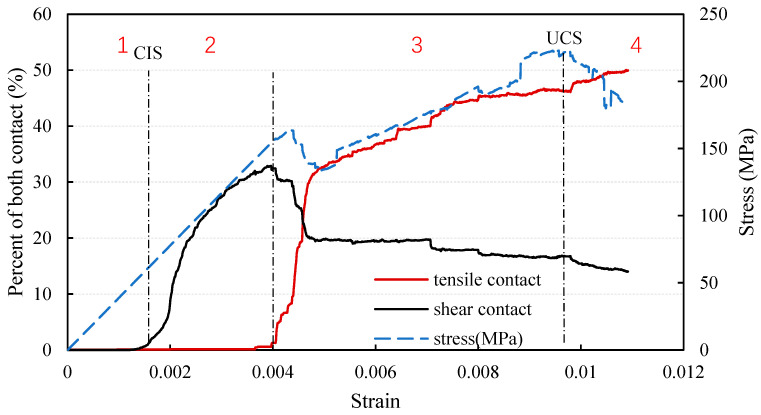
Relationship between shear cracks and tensile cracks for un-heat-treated material.

Figure 12 shows the percentage variation of shear cracks and tensile cracks during uniaxial compressive loading of un-heat-treated granite. The included stress–strain curve can be used to help explain the different stages. The predicted curve for the uniaxial compression test from UDEC is different from the experimental curve recorded in the laboratory. This difference can be understood in terms of shear contacts and tensile contacts. During the first stage of loading in the model, the stress increases linearly and there is essentially no change in the shear and tensile contacts. This stage corresponds to the experimental compaction stage and part of the elastic loading stage. In the second stage, the number of shear failures increases dramatically, but the number of tensile contacts does not change. Stage 2 corresponds to the experimental elastic loading stage. At the beginning of stage 3, tensile cracks begin to appear and the number of shear cracks declines. In this stage in the model, some of the shear cracks are subjected to tensile forces and thus convert to tensile cracks. After a sudden change in the number of shear and tensile cracks, the percentages of shear and tensile contact failures become more stable until final failure of the specimen. Stage 3 corresponds to unstable fracture development. The addition of a thermal cycle step in the calculation results in a longer unstable fracture development stage (see Figure 6). This feature is not obvious in the experimental stress-strain curves but is evident in the UDEC predictions. At the end of stage 3, the specimen fails. The applied stress decreases and the number of tensile cracks increases. It is clear from Figure 12 that the number of shear cracks starts to increase rapidly at the crack initiation stress (CIS) of 59.9 MPa which is 26.8% of the UCS. The number of shear cracks reaches a maximum, corresponding to 32.8% of all contacts, at the boundary between stages 2 and 3.

As a result, there is a shift in the kind of failure represented in the model, where shear contacts become tensile contacts. This result is in general agreement with the results of Kazerani (2013) and Gao (2013) who used triangular meshes in UDEC, although their results were slightly different [1,27]. However, it does not agree with the results of Nicksiar and Martin (2014) who observed shear cracks appearing later than tensile cracks [23].

**Figure 13:**
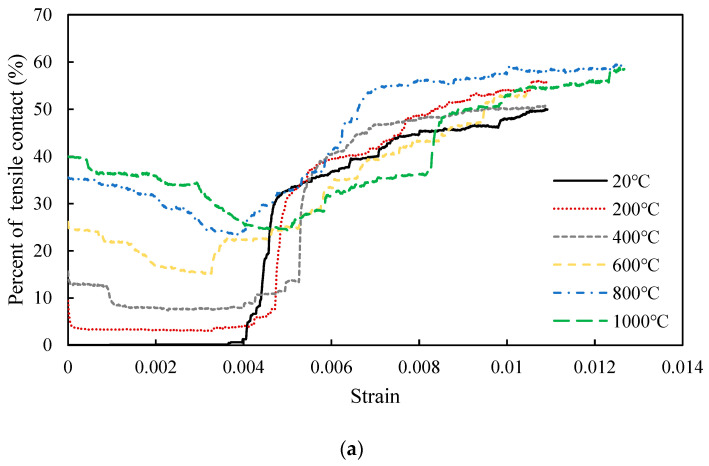

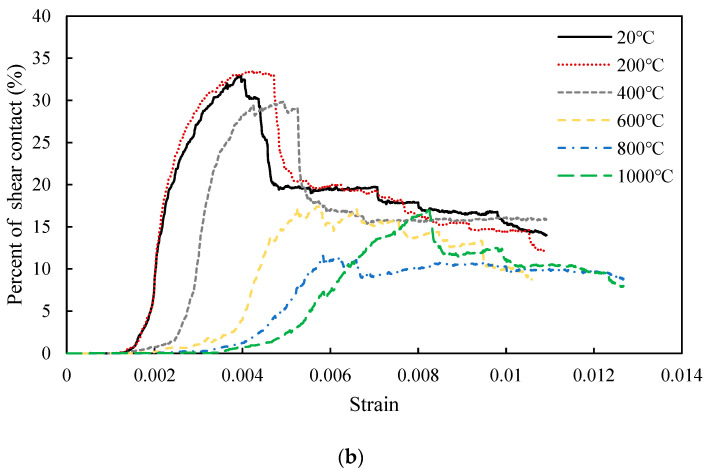
Percent of contact after being heat-treated with different temperature: (**a**) tensile contact; (**b**) shear contact.

To investigate the effect of heat treatment on the development of shear cracks and tensile cracks, the percentage of tensile contacts as a function of strain during uniaxial compression testing after heat treatment at different temperatures is shown in Figure 13a. The initial number of tensile cracks increases with the temperature of the modelled thermal cycle, with the highest value of 40% occurring after heat treatment at 1000 °C. It is a thermal expansion which causes the observed increase in the initial number of tensile contacts. At the beginning of the uniaxial compression test, the number of tensile cracks decreases in the specimens which have been exposed to high temperatures. Some of the tensile contacts close and then open again as the specimen enters the unstable fracture development stage. Examination of Figure 8 shows that the tensile contacts normal to the direction of the applied load close much more easily than the tensile contacts in line with the direction of the applied load during initial loading. Compared with the 20 to 400 °C curves, the curves corresponding to 600 to 1000 °C reach the unstable fracture development stage at a lower strain.

Figure 13b shows the percentage of contacts which have failed in shear as a function of strain during uniaxial compression testing after exposure to different temperatures. As the temperature of the thermal exposure increases, the number of failed shear contacts decreases. The number of contacts which have failed in shear in the 20 to 400 °C models increases dramatically during the elastic loading stage. As mentioned previously, with the new failure criterion employed in this study, if contact is subjected to tensile loading, it is no longer considered a shear crack. When the heat treatment temperature exceeds 600 °C, the rate at which the number of failed shear contacts increases becomes slower and the maximum number of failed shear contacts after thermal exposure at 600 to 1000 °C is lower than the maximum number after thermal exposure at 20 to 400 °C. It can thus be inferred that thermal exposure causes a volume expansion and produces several tensile microcracks in the material.

Although UDEC provides its own embedded criterion, users can create their Fish function to determine contact failure types. Based on the judging criterion contained in UDEC, a modified criterion, see Equation (2), was developed and a Fish function was written to extract information about shear and tensile contact failures. However, to verify whether the initial cracking which occurs during the uniaxial compression of granite is due to shear or tensile loading, additional numerical simulations may be required. With the criterion established in this paper, shear cracks rather than tensile cracks appear first. Furthermore, it was found that heat treatment affects the development of shear cracks and tensile cracks, and the transition of shear cracks to tensile cracks.

## 5. Conclusions

A numerical model for describing rock behavior under uniaxial compressive loading after heat treatment was established using the Voronoi method in UDEC. The model predictions were compared with the experimental results previously obtained by Chen et al. (2017) and an acceptable agreement was achieved. A modified criterion was proposed to differentiate between shear cracks and tensile cracks. From the results of these numerical investigations, the following conclusions can be drawn:

(1) If the material has a non-negligible high porosity, the porosity needs to be considered in the UDEC modelling process.
(2) In this research, the model established by Voronoi method can obtain the result which is more consistent with the actual situation after the thermal-mechanical coupling operation. Meanwhile, this method can be used to study the evolution or rock crack after thermal treatment.
(3) According to the modified contact statistics method, shear contact first occurs in granite specimen, and part of shear contacts turns into tensile contacts when the shear contact approaches the peak value.
(4) The thermal effect will lead to the advance of tensile contact. The higher the treatment temperature is, the higher the initial number of tensile contact is. The shear contact has the opposite rule.

## Figures and Tables

**Table 1:** Mechanical parameters used in model before and after calibration.

Block Parameter	Before	After
Density (kg/m^3^)	2724	2724
Poisson’s ratio	0.23	0.23
Young’s modulus (GPa)	40.23	30.23
Bulk modulus (GPa/m)	24.82	18.66
Shear modulus (GPa/m)	16.35	12.29

**Table 2:** Mechanical parameters for model joints before and after calibration.

Contact Parameter	Before	After
Joint bulk modulus (GPa/m)	186,520	20,000
Joint shear modulus (GPa/m)	186,520	16,000
Joint cohesion (MPa)	40	55.5
Joint residual cohesion (MPa)	0	0
Joint friction (°)	55	52
Joint residual friction (°)	45	35
Joint dilation (°)	0	35
Joint tension (MPa)	20	5
Joint residual tension (MPa)	0	0

**Table 3:** Mechanical parameters from experiments.

	1000 °C Experimental Result	1000 °C Thermo-Mechanical Result
Density (kg/m^3^)	2724	2724
Young’s modulus (GPa)	2.58	30.23
Bulk stiffness (GPa/m)	-	18.66
Shear stiffness (GPa/m)	-	12.29
Conductivity (W/(m^2^·K))	-	3.49
Specific heat (J/(kg·°C)	-	920
Thermal expansion coefficient (1/°C)	-	3 × 10^−6^
Joint bulk stiffness (GPa/m)	-	20,000
Joint shear stiffness (GPa/m)	-	16,000
Joint cohesion (MPa)	-	55.5
Joint residual cohesion (MPa)	-	0
Joint friction (°)	-	52
Joint residual friction (°)	-	35
Joint dilation (°)	-	35
Joint tension (MPa)	-	5
Joint residual tension (MPa)	-	0

## Data Availability

Not applicable.
